# Engineering In Situ Weldable Vascular Devices

**DOI:** 10.3390/bioengineering10020221

**Published:** 2023-02-07

**Authors:** Daniel Cohn, Fany Widlan, Matt Zarek, Ziv Peselev, Allan Isaac Bloom

**Affiliations:** 1 Casali Center of Applied Chemistry, Institute of Chemistry, The Hebrew University of Jerusalem, Jerusalem 9190401, Israel; 2 Vascular and Interventional Radiology, Hadassah-Hebrew University Medical Center, Jerusalem 91120, Israel

**Keywords:** in situ welding, polyurethane, abdominal aortic aneurysm, stent graft

## Abstract

(1) Background: The minimally invasive implantation of medical devices is largely limited by their insertion profile, and, therefore, minimizing them constitutes a leading trend in the field. (2) Methods: This study introduces the in situ welding strategy, whereby the components of the stent grafts used to treat abdominal aortic aneurysms were decoupled, deployed sequentially, and welded together at the aneurysmal site, greatly reducing their insertion profile. Polyurethane elastomers were used to produce the graft and to coat the metallic struts of the stent to render it in vivo weldable. Results: The composition of the polyurethanes was fine-tuned, so to minimize the insertion profiles and optimize the welding properties and the clinical performance of the devices assembled. The stent and graft were deployed successively in pigs via a small 8F introducer, in situ welded, and the patency of the bi-component device was confirmed over a three-month post-implantation period. The strength of the stent/graft welded connection was fully retained, with no de-welding observed. Conclusions: The in situ welding strategy resulted in implantations that were easier to perform and markedly less injurious to tissues and organs, largely expanding the applicability of these ultra-minimally invasive procedures to especially frail segments of the population.

## 1. Introduction

There are a mounting number of endoluminal devices that are currently available for minimally invasive procedures [1,2,3,4,5,6]. One of the key driving forces guiding the development of new endoluminal devices is the minimization of their insertion profile, which will result in implantation procedures that are markedly less injurious to tissues and organs. Furthermore, this strategy will not only broaden the use of these devices but will also improve their clinical performance. This study introduces a strategy to markedly reduce the insertion profile of endoluminal devices by separating their components, deploying them sequentially, and rapidly and securely welding them together at their site of performance. Even though this approach is of broad clinical applicability, such as along the vasculature, in the airway, or the GI tract, this concept is illustrated here by the treatment of Abdominal Aortic Aneurysms (AAA).

The formation of aneurysms is a dangerous degenerative pathology of arterial tissues, especially common in adult and elderly populations, whereby the wall of the artery weakens locally and expands, often resulting in vessel rupture [7,8,9,10]. The first endoluminal device, consisting of a vascular graft sewn to a metallic stent and deployed at the aneurysmal site using a saline-filled balloon, was introduced in 1991 with the purpose of isolating the compromised vessel wall from blood flow [11]. These bi-component devices are called stent grafts. While this technique, named Endovascular Aneurysm Repair (EVAR), represented a breakthrough compared to the open abdomen repair, currently, only about 60% of AAA patients are eligible for this procedure [12,13,14,15,16,17]. The most important shortcoming limiting the clinical use of EVAR devices pertains to the presence of the fabric in the stent grafts that more than doubles the diameter of the bare stent and renders the bi-component device bulky and rigid. Their large profile and significant stiffness constitute major drawbacks of contemporary stent grafts [18] since they cannot be deployed through narrow, tortuous, or calcified access vessels [19,20,21,22,23,24,25], primarily the iliac arteries.

Aiming to overcome the serious drawbacks of existing stent grafts, herein we introduce an in situ welding approach, whereby we sequentially deploy the metallic stent and then the polymeric graft, which are then welded together at the aneurysmal site. By doing so, the insertion profile of the device is dramatically reduced, and its flexibility is largely increased. It is consensually agreed that the fabric occupies at least 60% of the cross-section of the stent grafts currently in clinical use. Since, by definition, each of the device elements—the stent and the graft—are substantially smaller than the bi-component stent grafts, deploying them sequentially reduces by at least half their insertion profile.

In the frequent cases when the iliac arteries are also compromised, and two or more endografts are needed, this strategy also enhances the stability of the modular device and precludes the risk of leakage between the stent grafts, by strongly and hermetically welding them together.

The in situ welding approach also facilitates customization of the endografts so they comply with the specific requirements posed by the anatomy of each individual patient. Furthermore, in situ weldable ultrathin polymeric components, which we named sleeves, that substitute the thick textile grafts currently used were developed and resulted in additional profile reduction. Since metals cannot be welded at clinically relevant temperatures, they were rendered in vivo weldable by coating them with an appropriate polymer.

Two different types of polyurethane elastomers were used to generate both the sleeve and the coating of the struts of the metallic stent. The soft segment of the polyurethanes synthesized consisted of a polyether (polytetramethylene glycol) or a polyester (polycaprolactone) of different molecular weights chain extended in all cases with hexamethylene diisocyanate (HDI), whereby polyether urethanes or polyester urethanes were formed, respectively.

## 2. Materials and Methods

### 2.1. Synthesis and Characterization of the Polyether Urethane Elastomers

Polytetramethylene glycol (PTMG) (650, 1000, and 2000 g mol^−1^) was obtained from BASF, while hexamethylene diisocyanate (HDI) and stannous octoate (SnOc) were purchased from Sigma Aldrich and used as received. Chloroform, dioxane, and petroleum ether were purchased from BioLab, Ashkelon, Israel, and used as received, except dioxane, which was dried over 4 Å molecular sieves for 24 h.

The synthesis of the polyether urethane polymers is exemplified here for the polymer comprising PTMG 650 segments. An amount of 30 g of 650 g mol^−1^ PTMG diol was dried for 2 h at 120 °C under vacuum with stirring. Under nitrogen, the temperature was lowered to 85 °C, a mechanical stirrer was introduced, and 140 μL of SnOc was added. Subsequently, 8.9 mL of HDI was added dropwise, and as the viscosity increased, 5 mL aliquots of dry dioxane were added. After no additional increase in the viscosity was observed, 200 mL of chloroform was added, and the solution was precipitated in petroleum ether. The precipitate was dried overnight under a vacuum at room temperature. These polymers are denoted PTUR, followed by a number that designates the molecular weight of the PTMG segment; then, the chain extended with HDI.

### 2.2. Synthesis and Characterization of the Polyester Urethane Elastomers

Polycaprolactone (PCL) diol (1250, 2000, and 14,000 g mol^−1^), hexamethylene diisocyanate (HDI), and stannous octoate (SnOct2) were purchased from Sigma Aldrich, St. Louis, MO, USA and used as received. The PCL-diol (3000 and 4000 g mol^−1^) was obtained from Perstorp (Malmö, Sweden) as a gift. Methanol, chloroform, dioxane, and petroleum ether were purchased from BioLab, Israel, and used as received, except dioxane, which was dried over 4 Å molecular sieves for 24 h.

The synthesis of the polyester urethane elastomers is exemplified here for the polymer comprising PCL 3000 segments. An amount of 40 g of 3000 g mol^−1^ PCL diol was dried for 2 h at 120 °C under vacuum with stirring. Under nitrogen, the temperature was lowered to 85 °C, a mechanical stirrer was introduced, and 42 μL of SnOct2 was added. Subsequently, 2.6 mL of HDI was added dropwise, and as the viscosity increased, 5 mL aliquots of dry dioxane were added. After no additional increase in the viscosity was observed, 200 mL of chloroform was added, and the solution was precipitated in petroleum ether. The precipitate was dried overnight under a vacuum at room temperature. These polymers are denoted CLUR, followed by a number that designates the molecular weight of the PCL segment; then, the chain extended with HDI.

The molecular weight, polydispersity, and degree of polymerization of the synthesized polymers were determined by Gel Permeation chromatography (GPC)-Waters 2690 with a Differential Separations Module, Styragel columns, and chloroform eluent.

### 2.3. Preparation and Analysis of Polymer Sleeves

To prepare the sleeves, glass tubes with external diameters of 12 mm and 7 mm, corresponding to the aortic and iliac arteries of a pig, respectively, were dip coated in an 8% (*w*/*w*) chloroform solution of CLUR using a customized dip coater. Immersion in methanol was used to remove the sleeves from the glass tubes. The morphology of the polymers was determined using a Differential Scanning Calorimeter, Mettler TA-400, at a heating rate of 10 °C/min, from 0 °C to 100 °C, and under a nitrogen atmosphere. The mechanical properties of the sleeves were measured using a Universal Instron Testing Machine Model 1114 at a 10 mm min^−1^ cross-head rate. The burst pressure of the sleeves was measured using a pressure chamber as described [26]. The sleeves were cut to 3 cm long, marked at each end with DuPont silver paste 5025 to render the sleeves radiopaque, and left in an oven at 37 °C for 24 h.

### 2.4. Cytotoxicity Testing

A standard cytotoxicity test of a CLUR4k film (test device) was carried out at Harlan Laboratories, Rehovot, Israel. The test device positive control (0.1% Zinc Diethyldithiocarbamate (ZDEC) in polyurethane film) and negative control (glass vial) were extracted with L929 growth medium. Each one was cut into small pieces and extracted in L929 Growth Medium in a 6 cm^2^/mL ratio at 37 ± 1 °C for 72 ± 2 h with slow agitation. The extracts were diluted for cell treatment with L929 growth medium at serial dilution to 100%, 85%, 75%, 50%, 25%, and 12.5% (six replicates of each). L929 line cells were exposed to the diluted extracts and incubated at 37 ± 1 °C for 24 ± 2 h.

### 2.5. Preparation of the Bi-Component Endovascular Device

Powerflex Pro balloon catheters of dimensions 7 mm × 6 cm and 12 mm × 6 cm (cat No. 4400706S, 4401206S), as well as Palmaz Genesis balloon expandable stents (PG395P, PG3910P), were purchased from J&J, Israel. The bare metal stents were dip coated in an 8% (*w*/*w*) chloroform solution of CLUR4K or PTUR650.

The sleeves with the radiopaque markers were attached to the balloon catheter with 2 µL of a 10% poly-N-vinyl pyrrolidone (PVP) 40 kDa solution in methanol, tightly wrapped around the balloon and sealed with 10 µL of the same PVP solution. The PVP polymer was purchased from Sigma Aldrich and used as received.

Both the coated stents and the sleeves attached to the balloon catheters were subjected to a low temperature (31.5 ± 3 °C) ETO sterilization cycle (480 min exposure) at Mediplast Ltd., Yavne, Israel, followed by a 140 min venting step.

The strength of the welding connection between the sleeve and the coated metallic stent was measured by means of a pull-out experiment using a Universal Instron Testing Machine Model 1114, at a 10 mm min^−1^ cross-head rate and with a Lutron Tensiometer FG-5000A.

### 2.6. In Vivo Studies

Animal implantations were carried out at the Animal Surgery research facility of the Hadassah University Medical Center, following approval from the Ethics Committee for the care and treatment of laboratory animals, Hebrew University of Jerusalem (Research project number: MD-12-13541-4, NIH approval number: OPRR-A01-5011). The study included a total of 11 pigs, with the first two performing as controls using commercially available devices for survival periods of up to 3 months. A female pig (average weight: 55 kg) was placed under general anesthesia, and with ultrasound guidance, the bilateral femoral arteries were accessed with commercially available vascular access devices (8 French Bright Tip vascular access sheath). The animals received 100 mg of Aspirin daily and were fully heparinized. Catheter angiography of the abdominal aorta and pelvic arteries was performed to verify positioning. A sterile thermocouple was positioned in the vessel to measure baseline and deployment temperatures by inserting it via one of the vascular access sheaths. A Palmaz Genesis stent coated with CLUR4K was hand crimped onto the Powerflex angioplasty balloon, which was approximately 2 cm longer than the stent, to prevent slippage of the stent from the balloon during deployment. The stent was inserted using continuous fluoroscopic guidance, one in the distal abdominal aorta (inflated to a pressure of 4.5 atm to achieve a nominal diameter of 12 mm) and the other in the common iliac artery (inflated to a nominal pressure of 8.0 atm suitable for 7 mm diameter). Following successful deployment, confirmed angiographically, the balloon catheter was removed. Subsequently, the 3–4 cm long sleeve, pre-mounted on another Powerflex angioplasty balloon, was inserted through the same introducer, navigated to the site fluoroscopically, and aligned with the stent fluoroscopically using the radiopaque markers. After 4 min, the time previously determined for the PVP adhesive to dissolve, the balloon was inflated with hot saline mixed with aqueous contrast material (ratio of 1:5) to achieve adequate radio-opacity during balloon inflation and deflation. The hot water/contrast material mixture was aspirated into a pressure inflation device which was then rapidly attached to the balloon catheter for inflation and sleeve welding. The balloon was inflated to a nominal pressure of 4–8 atm depending on location, deflated after 60–90 s, and easily removed with no sleeve residual material attached to it, while the marker positions were evaluated angiographically. Following the removal of the balloon catheter, repeat angiography was performed to verify adequate welding of the sleeve to the stent. If acute, at the termination of the experiment, the animal was euthanized according to institutional guidelines, and the investigational device was surgically explanted for further examination. The specimen was rinsed in water and immediately placed in formalin for preservation and analysis.

## 3. Results and Discussion

### 3.1. The In Situ Welding Working Concept

The conceptual cornerstone of this study aims at markedly reducing the insertion profile of endoluminal devices by implanting their different components successively and rapidly welding them together at their site of performance. Regardless of the anatomical site of intervention, e.g., the tracheo-branchial tree, the GI tract, the urinary system, or along the vasculature, this approach results in a remarkable decrease in the profile of insertion of the device, largely expanding the population eligible for these minimally invasive procedures.

In polymer welding, two components are brought to contact, and molecular mobility is induced by heat or a suitable liquid. As the molecules near the surface undergo rearrangement, wetting, and diffusion, the interface gradually disappears with the increase of intersecting chains, and the mechanical strength of the welded connection increases, approaching the cohesive strength of the bulk [27,28]. The strength of the welded connection is a function of the welding time and temperature, the contact pressure, the molecular weight (including polydispersity), and the nature of the polymer [29,30].

**Figure 1:**
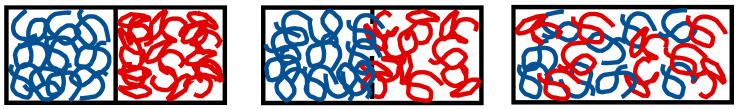
Schematic depiction of the welding phenomenon over time until the disappearance of the interface.

Figure 1 schematically depicts the welding phenomenon, showing the spatial reorganization of the macromolecules close to the surface contacting the two polymeric objects. As shown, as the chains intersect the interface, they gradually result in its disappearance, in optimal scenarios regenerating in the welded connection the cohesive strength of the polymer.

The welding temperature, Tw, is defined hereby as the lowest temperature at which the interface disappears, and the strength of the welded connection equals that of the bulk within less than 30 s. Due to obvious clinical constraints, it is critical for the in situ weldable polymer to exhibit a low softening temperature so that Tw is physiologically acceptable. An angioplasty balloon filled with suitably warm saline provides both the temperature and pressure required.

### 3.2. Synthesis, Characterization, and Performance of Weldable Polymers

**Figure 2:**
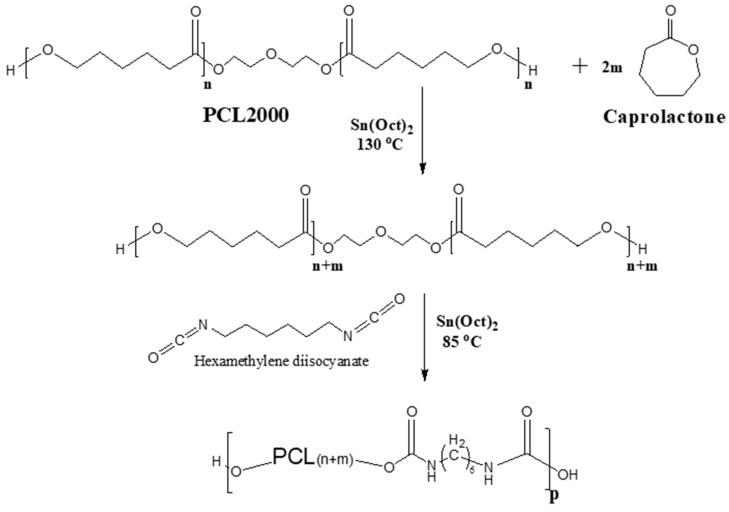
The reaction scheme of CLUR synthesis.

In order to weld together the metallic stent and the polymeric sleeve in vivo, the struts of the stent were coated with a thin layer of the same polymer used for the polymeric sleeve. To generate both the sleeve and the strut coating, two families of polyester and polyether urethane elastomers comprising semi-crystalline polycaprolactone (PCL) (CLURs) or polytetramethylene glycol (PTMG) segments (PTURs), respectively, were synthesized. Both polymers were synthesized by the equimolar reaction of the different hydroxy-terminated PCL or PTMG segments and hexamethylene diisocyanate (HDI). The reaction scheme is illustrated in Figure 2 for a CLUR polymer. Since the crystallizability of semi-crystalline polymers correlates with their molecular weight [29,30,31], the length of the soft segments, PCL or PTMG, was tightly controlled, and the melting point and degree of the crystallinity of the resulting polymers were fine-tuned. As a result, their Tw was also optimized, as dictated primarily by clinical considerations.

Even though PCL is a known biodegradable polymer [32,33,34], it was used as a model material to demonstrate the in vivo welding concept due to the ability to closely control the molecular weight of the PCL diol chains by the ring opening polymerization of ε-Caprolactone. It should also be stressed that PCL degrades extremely slowly over a period of several years, and, therefore, its degradability is immaterial compared to the time scale of the present study [35].

**Figure 3:**
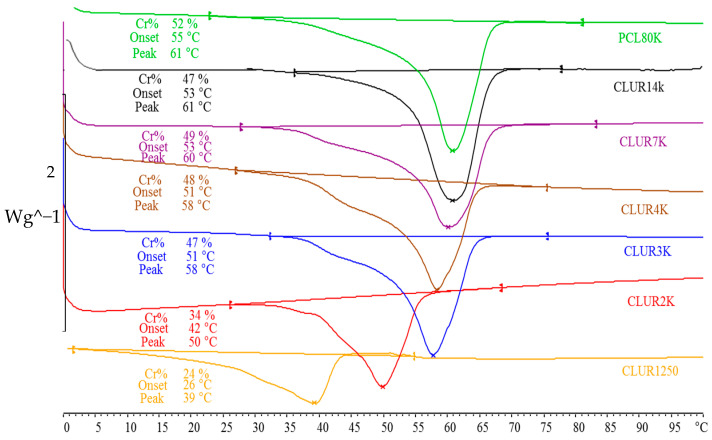
DSC curves of CLUR polymers based on PCL segments of different molecular weight.

In accordance with theory [34], the set of CLUR polymers synthesized exhibited a gradual increase in their degree of crystallinity and melting temperature (Tm) with molecular weight. The Tm values spanned from 39 °C, when the CLUR polymer comprised PCL1250 g mol^−1^ segments, to 61 °C for PCL 80,000 g mol^−1^ segments (Figure 3).

**Figure 4:**
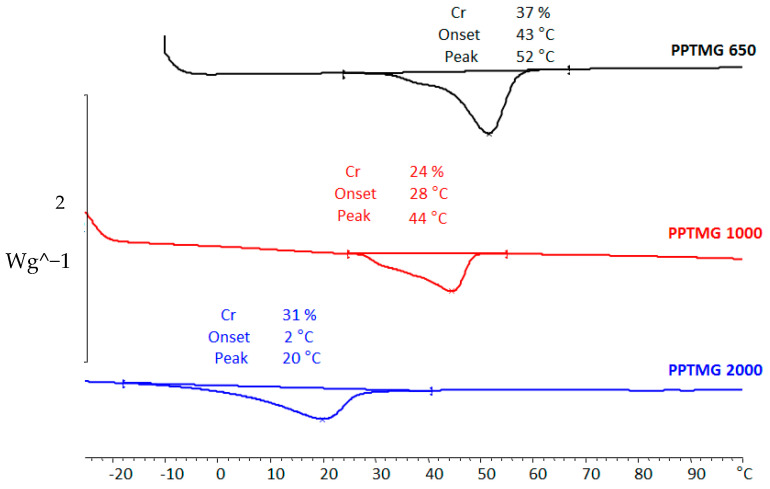
DSC curves of PTUR polymers based on PTMG segments of different molecular weight.

Also, PTMG-based thermoplastic polymers synthesized by the equimolar reaction of PTMG diols of different molecular weights and HDI were investigated. The thermograms shown in Figure 4 reveal that PTUR 1000 and PTUR 2000 melt, substantially or fully, at temperatures below 37 °C and are, therefore, unsuitable for in vivo conditions. The fact that the PTMG chains melt at a higher temperature, the shorter they are, is due to their higher content of urethane groups able to generate strong inter-molecular hydrogen bonds, as their molecular weight is smaller. PTUR 650, on the other hand, melted at a suitable supraphysiological temperature and was chosen for further study.

**Figure 5:**
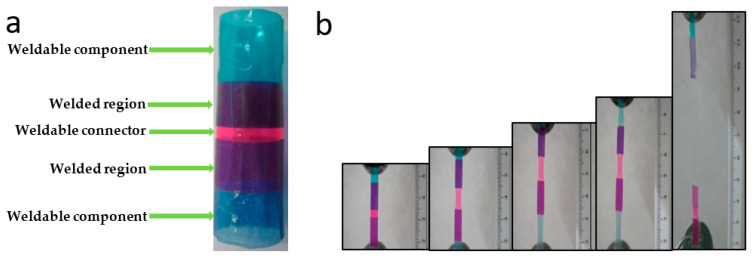
(**a**) Two PTUR650 short sleeves (blue) were welded together in vitro at 43 °C using a connector (pink) made of the same polymer; (**b**) Tensile test of strips taken from the welded sleeves.

The excellent weldability of PTUR650 was demonstrated by the experiment shown in Figure 5, where two PTUR650 short sleeves (blue) were welded together in vitro using a connector (pink) made of the same polymer (Figure 5a). The welding procedure was performed within a Tygon tubing using a balloon filled with water at 43 °C and a welding time of 20 s. With the aim of determining the strength of the welded connection, three longitudinal strips were cut from the welded sleeves and subjected to tensile testing (Figure 5b), which displayed a stress at break of 13 ± 4 MPa and a tensile modulus of 227 ± 1 MPa. It is worth stressing that the failure was always cohesive, without any de-welding between the sleeves being observed.

Most of the work focused on CLUR polymers because of the enhanced ability to fine-tune their thermal and superior mechanical properties.

CLUR polymers displayed very good mechanical properties and formed strong films effortlessly, all of them displaying stress at break values greater than 30 MPa. The gradually increasing degree of crystallinity of CLUR polymers, generated by progressively longer PCL segments, resulted in sleeves exhibiting different levels of stiffness, with tensile moduli rising from 35 MPa for CLUR1250, to 350 MPa for CLUR14K. CLUR4K was selected as the in situ weldable polymer because of its optimal combination of mechanical properties (σ_F_ = 20 ± 3 MPa, E = 232 ± 9 MPa, ε_F_ = 1543 ± 100%) and T_w_ = 45 °C.

Thin sleeves, 15 ± 5 µm thick, were formed by dip coating the polymer solution on glass rods with 7 mm and 12 mm outer diameters that matched the average inner diameter of the aorta and iliac arteries of the porcine model, respectively, as described in the Materials and methods section.

**Figure 6:**
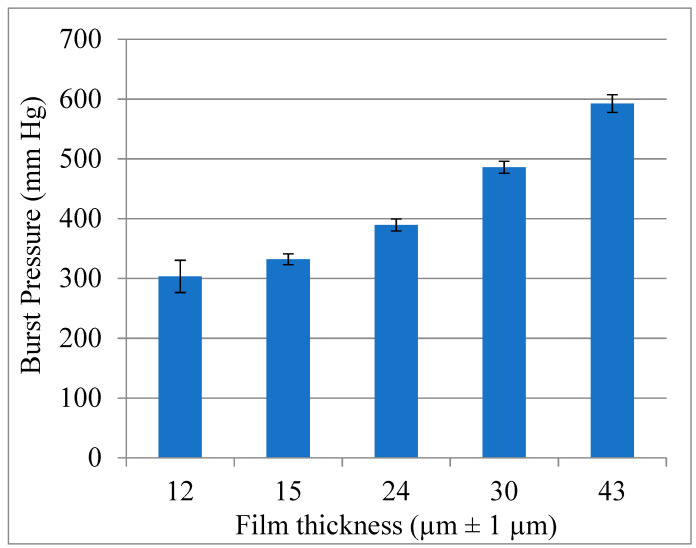
Preparation of the sleeve. Burst pressure of thin CLUR film (error bars are SD, n = 5).

In order to assess the capacity of the polymer to withstand the pressure of aortic blood flow, the burst pressure of 1 cm^2^ films was measured for different film thicknesses. The measurements were performed using a pressure chamber connected to a manometer and a pressurized air inlet, as described by Carbon et al. [26]. Figure 6 shows that even a film as thin as 12 µm exhibited a burst pressure of 300 mmHg, which ensures the ability of the sleeve to sustain also very high blood pressure levels [36,37,38]. Furthermore, since the area between the struts is much smaller than the 1 cm^2^ dimensions used in this study, even thinner sleeves could be used to span between the struts of the metallic stent.

**Figure 7:**
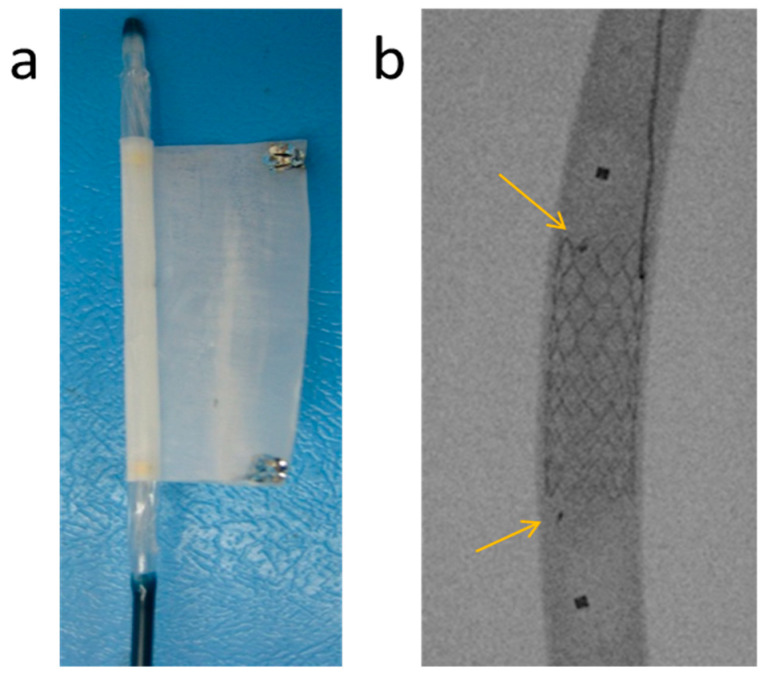
(**a**) Silver markers were placed at the edges of the CLUR sleeve after being mounted on an expandable angioplasty balloon before wrapping it around the balloon; (**b**) Angiographic view of a sleeve welded to the stent inside a Tygon tube using an expandable balloon. The arrows point at the silver markers on the sleeve, while the squares correspond to the balloon markers.

Similar to the coatings used in drug-eluting stents [39,40], the struts of the metallic stent were coated with a 10 ± 5 μm thick layer, rendering the stent in vivo weldable to the sleeve. Since the EVAR procedure was conducted under fluoroscopic monitoring and, seeking to visualize the position of the polymer sleeve, two small radiopaque markers were placed at the ends of the sleeve using a silver paste (Figure 7a). The markers are visible at the ends of the sleeve while being deployed by the angioplasty balloon and being welded to the pre-coated stent, as shown in Figure 7b.

Due to their relatively low Tm values, the sleeves and coated stents were sterilized with a low temperature (31 °C) ethylene oxide cycle to avoid risking deforming the sleeve. Macroscopic inspection of the sleeves as well as analysis of their molecular weight and mechanical properties prior to and following sterilization, conclusively demonstrated that sterilization had no measurable effect on the sleeves (Data not shown).

The cytotoxicity of CLUR films was assessed by extracting them in L929 Growth Medium at 37 °C for 72 h, and after appropriate dilution, L929 line cells were exposed to the diluted extracts and incubated at 37 °C for 24 h. The findings showed extremely high viability values of the cells (close to 100%) for all the extract percentages of the polymer film in the growth medium, indicating that they are not cytotoxic.

### 3.3. Pre-Implantation Performance of the Bi-Component Device

**Figure 8:**
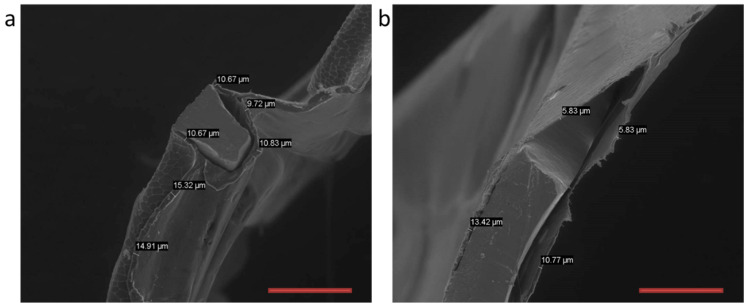
SEM micrographs of the stent: (**a**) Strut cross-section of the crimped stent; (**b**) Strut cross-section of the expanded stent. The thickness of the coating prior to and following expansion is also shown. (Scale bar: 200 μm.).

The sleeve was mounted on the catheter and tightly wrapped around it, whereby a very low profile was obtained. Seeking to ensure that it will not slip off during the insertion and navigation stages of the procedure, in addition to its tight wrapping around the angioplasty balloon, the sleeve was also temporarily secured with a water-soluble adhesive, polyvinyl pyrrolidone (PVP). After navigating the sleeve to the aneurysmal site, PVP dissolved quickly as a result of its exposure to blood, allowing the sleeve to be easily unwrapped as the balloon was inflated with warm saline. The sleeve was then rapidly welded to the coated stent (in less than 20 s), while the sleeve’s outer surface temperature was registered at 45 °C. The metal stent was dip coated in an 8% *wt*/*wt* chloroform solution of CLUR. The SEM micrographs in Figure 8a (crimped stent) and Figure 8b (expanded stent) focus on the region where coated stent struts were intentionally cryo-cleaved under liquid nitrogen, showing the cross-section of the metal strut and its polymeric coating.

As measured during the SEM study, the average thickness of the coating decreased from 10–15 μm before expansion to 5–10 μm after expansion. As shown by the micrographs, due to the strength, flexibility, and high elongation at the break value of CLUR, the coating did not tear, and the struts remained fully coated by the polymer, also when the stent was fully expanded. The welded connections between the coated struts and the sleeve rely, therefore, on the high cohesive strength of the polymer and not on the weak adhesion forces between the metal strut and the polymeric coating.

**Figure 9:**
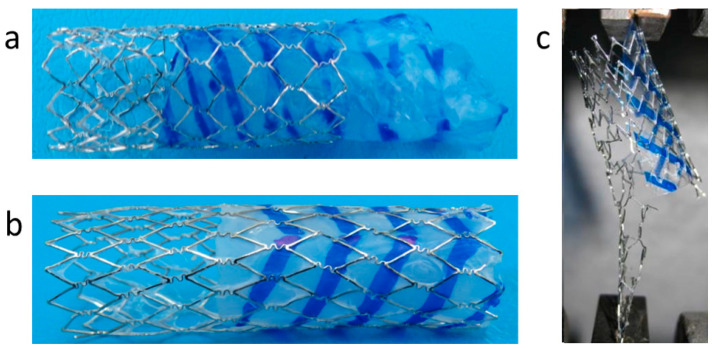
In vitro test of welding strength: (**a**) Image of the sleeve welded to the coated stent, using CLUR4K; (**b**) The sleeve after the pull-out test. (**c**) Pull out test of a PTUR650 patch welded to the covered stent.

As reported by Liffman et al. [41], blood flow exerts an average shear force of 10 N on the aortic wall, which the device must resist. An in vitro pull-out force measurement performed on a sleeve welded to a coated stent using the Instron resulted in a force of 18 ± 2 N that caused the sleeve to fail cohesively but without de-welding from the coated stent (Figure 9a,b). It is worth stressing that the pull-out test conducted is substantially harsher than the mechanical regime under which the device is called upon to perform in vivo. The same result was obtained when evaluating the welding of a PTUR650 patch to a stent coated with the same polymer. Figure 9c compellingly demonstrates the strength of the welded connections by showing that it was the metallic stent that broke while the weld connection stood in place.

### 3.4. In Vivo Evaluation of Bi-Component Device

The feasibility of the in situ welding technology was evaluated in vivo using a porcine model. Under general endotracheal anesthesia, both femoral arteries were accessed using standard angiographic techniques, and 8 French (F) (one French is a third of a millimeter) vascular introducer sheaths were inserted bilaterally. After hand crimping partially expanded stents that were coated with CLUR4K onto a standard angioplasty balloon, they were inserted and deployed under fluoroscopic guidance.

**Figure 10:**
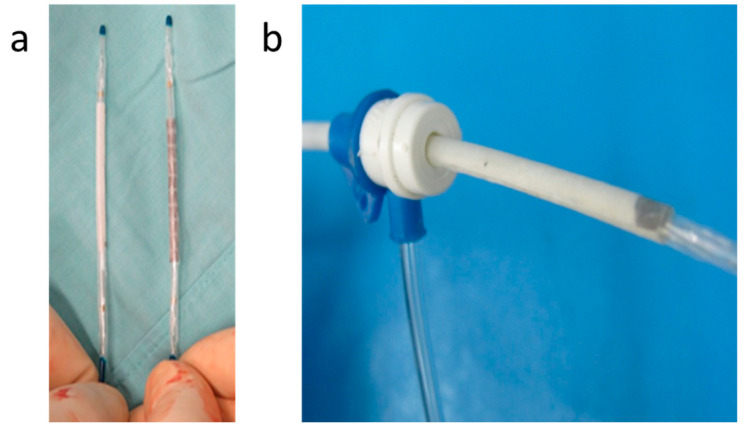
In situ welded endovascular device preparation: (**a**) Sterilized sleeve (left) and coated stent (right) mounted on expandable balloons; (**b**) The device was easily inserted through an 8F vascular introducer sheath.

Figure 10a shows the pre-coated stent and its corresponding sleeve, both mounted on inflatable angioplasty balloons, prior to their implantation into the iliac artery. The insertion of both the stent and the sleeve was carried out through an 8F introducer sheath (Figure 10b) which proved to be smooth and straightforward. This demonstrates that the in situ welding strategy introduced in this work reduces the insertion profile by more than 50% since currently available devices require 14–18F introducers.

**Figure 11:**
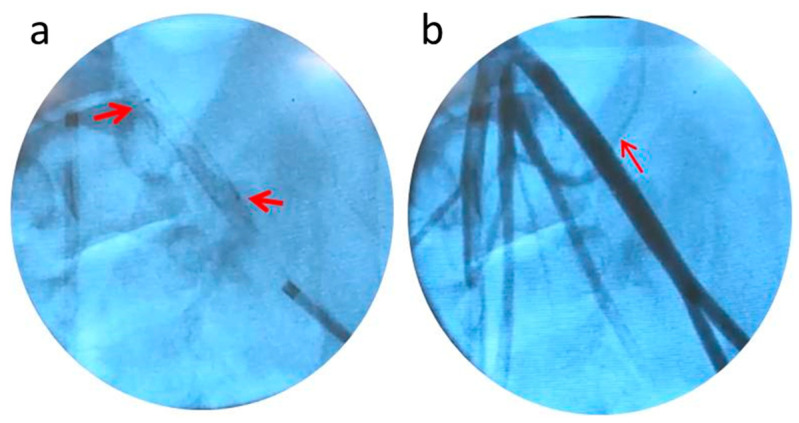
Deployed in situ welded endovascular device: (**a**) The polymer sleeve welded to the stent in the iliac artery of a pig. The arrows point to the radiopaque silver markers; (**b**) Flow of contrast agent through the lumen of the implanted device was undisturbed.

After alignment of the markers of the sleeve with the already implanted stent, the balloon was expanded with warm saline, welding the sleeve to the coated stent at 46 °C (as measured by the thermocouple inserted via the contralateral iliac artery) in 20 s (Figure 11a). The pressure required for rapid and strong welding was low when compared with pressure levels routinely used during endovascular procedures and typically fall in the 7–8 atmosphere range. The arrows in Figure 11a point at the sleeve markers. In Figure 11b, an angiogram reveals normal blood flow through the lumen of the in situ welded device.

**Figure 12:**
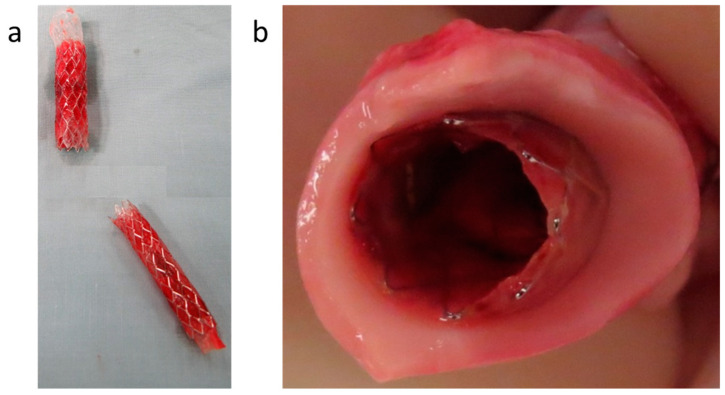
(**a**) Two explanted devices (upper device is 12 mm diameter and lower right is 7 mm diameter); (**b**) Explanted aorta with the implanted device after two weeks survival.

The same procedure was then performed for the 12 mm diameter stent in the aorta with the same ease and success. A 12 mm diameter stent was inserted using an 8F introducer and deployed in the infrarenal abdominal aorta using the same technique described above. The pre-mounted 3 cm-long sleeve was then welded to the coated stent, and successful welding was confirmed by intra-procedural angiography. Following the successful implantation, the pig was kept alive for 2 weeks, then imaged by CT angiography to verify the stent position and patency, as well as to look for any evidence of local vascular injury in response to the device. Figure 12a shows the 12 mm (upper) and the 7 mm (lower) in situ welded endografts after implantation in the aorta and iliac artery, respectively. The gross examination of the explanted devices shows, therefore, the successful in vivo welding of the sleeves to the stents, as well as the blood compatibility of the sleeve and the patency of the device. Figure 12b shows the explanted device of a 2-week survival implantation in a pig’s aorta. The polymeric sleeve and the coated stent were shown to be strongly welded. The same procedure was performed in another survival experiment, where after 3 months, the welding was found to be still in place, with both components strongly welded together. The aortic wall was peeled off with tweezers and mechanically separated from the device to show more clearly the intimate connection between the stent and the sleeve.

## 4. Conclusions

It has been shown that the strong and fast in situ welding of a polymer sleeve to a coated stent is a feasible strategy for the deployment of the ultra-low profile, flexible stent grafts. It was also demonstrated that the welding can be conducted successfully at a physiologically acceptable temperature and very rapidly (around 20 s). This was achieved by rationally tailoring the in situ weldable polymer so that its relevant thermal transition, Tg or Tm, is only slightly supra-physiological while displaying much enhanced mechanical properties at 37 °C. It can also be concluded that the polymer used to engineer the sleeve and coat the struts of metallic stent can be fine-tuned so that welding is accomplished using low pressures. The decoupling of the stent and the graft results in a remarkable reduction of the insertion profile, typically by half, when compared to current commercially available devices. The animal studies demonstrated the clinical feasibility and simplicity of this technique, enabling a large expansion of the applicability of EVAR procedures. Furthermore, it is anticipated that it will also be applicable to a variety of devices, such as in the tracheo-branchial tree, the vasculature, the urinary system, the GI tract, or the cardiac arena.

## Data Availability

All data generated or analyzed during this study are included in this article.
